# Vaccination Gap, Vaccination Fraud and Inefficient Testing: Lessons From the Coronavirus Pandemic

**DOI:** 10.1007/s10272-022-1072-3

**Published:** 2022-10-10

**Authors:** Hanno Beck, Aloys Prinz, Elmar Wolfstetter

**Affiliations:** 1 Economics, University of Applied Sciences Pforzheim, Tiefenbronner Str. 65, 75175 Pforzheim, Germany; 2 Institute of Public Economics II, University of Münster, Wilmergasse 6-8, 48143 Münster, Germany; 3 Institut für Wirtschaftstheorie I, Humboldt-University zu Berlin, Spandauer Str. 1, 10178 Berlin, Germany

**Keywords:** I12, I18

## Abstract

A number of shortcomings in Germany’s efforts to contain the spread of the coronavirus, including fraudulent testing, vaccination fraud and insufficient testing capacity have been identified and need to be remedied before another wave or worse, another pandemic. This paper examines the failures of the deterrence instruments and proposes solutions to address them.

More than two years ago, a pandemic erupted that is not yet over. After its first detection in Wuhan, China, a succession of new mutations of the coronavirus, ranging from the Alpha, to the Delta, to the Omicron variant, has kept the world in suspense.

After the development of effective vaccines, their large-scale deployment promised a speedy containment of the pandemic. However, many people have refused to be vaccinated while the rise of the more contagious Omicron variant accelerated the spread of infections. At the same time, in Germany we have seen that fraudulent testing, vaccination fraud and insufficient testing capacity, aggravated by poor testing methods, impaired the containment of the pandemic.

This paper discusses some failures of the containment strategy in Germany as well as possible solutions, inspired by economic analysis.

**Hanno Beck**, Pforzheim University, Germany.

### Vaccination gap

One of the most pressing issues to address in the containment of the virus is the vaccination gap. As too few people are vaccinated (see Figure 1), the spread of the virus is still out of control, leading to more severe infections and overcrowded hospitals.

People who are not vaccinated are more likely to be infected and will therefore have four kinds of side effects or externalities for others: they are more likely to spread the virus, they contribute to the production of new mutations of the virus, they impose a financial burden upon the health insurance system and they crowd out the treatment of patients with other health issues.

There are multiple reasons why people choose not to be vaccinated. One reason is the classical "free-rider problem" that plagues all vaccination programmes. A rational person may choose not to be vaccinated, because if many others are vaccinated, he or she shares the benefit of vaccinations without having the risk of potential side effects, no matter how small. If free riding is a frequent response and the health risk and its repercussions on the economy are as serious as they are in this pandemic, the reasonable public health response is to make vaccinations mandatory.

If one mandates vaccinations, one may use either a "carrot approach" or a "stick approach". The carrot approach offers financial or other rewards for those who consent to be vaccinated, while the stick approach penalises those who refuse vaccination. However, the carrot approach may induce speculation for ever higher rewards and may crowd out those who are otherwise

Figure 1

**Vaccinations per day in Germany**

30 days moving average

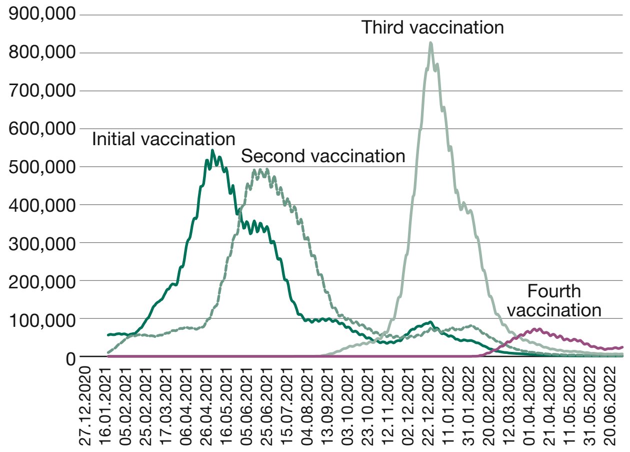

Source: RKI (2022), own calculations.

willing to be voluntarily vaccinated without financial reward.

A simple scheme to implement mandatory vaccinations could be as follows: Every adult resident is charged a lump-sum coronavirus tax that is billed in the annual income tax statement. That tax is refunded instantly when taxpayers file their income tax returns and supply proof of vaccination with their income tax statement. The net tax revenue can be used to subsidise the health insurance system or hospitals that were at the forefront of treating patients with COVID-19 infections. The appeal of this scheme is that one does not need to monitor peoples' vaccination status because that information is automatically forthcoming. This tax scheme could also be used to generate an anonymised data base on vaccinations that could automatically be deleted when the pandemic is over.

This tax scheme will most likely be effective because people tend to do everything imaginable to reduce their taxes. Distributing the net tax revenue to health insurers or hospitals could contribute to internalising the financial burden that the unvaccinated impose on others by overcrowding hospitals and taking advantage of the solidarity principle built into our health care systems.

## Fraudulent testing and counterfeit vaccination records in Germany

Another problem that needs to be addressed concerns the widespread fraudulent testing and counterfeiting of vaccination certificates.

### Coronavirus tests

In some countries rapid antigen coronavirus tests are provided free of charge on a large scale by testing centres, funded with public money. In Germany, these tests are widely used because a negative test result has been a prerequisite for entering shops or attending public events.[1] The operators of these test centres do not have to offer proof that they actually provided a test.[2]

This method of funding COVID-19 tests encourages fraudulent behaviour on an alarming scale and not only wastes taxpayer money, but also distorts the detection of infections, which is the *ultima ratio* for providing tests free of charge.

Insufficient or inadequate control mechanisms encourage fraud at testing centres (Beck et al., 2022).[3] The lack of control provides incentives for billing tests that were actually never performed, and test centres have no incentive to detect an infection – after all, that is what this mandatory testing is about. Moreover, not all test takers are interested in a correct test, but instead in a clean bill of health (e.g. to attend events). This favours sloppy testing and potential fraud even further, as the tested person is no longer a control authority. On top of that, the current practice induces the employment of unskilled staff and maximises the testing centre's revenue rather than test quality.[4]

A simple proposal to stop fraud at testing centres is to pay only positive tests instead of paying every test. If a rapid test shows a positive result that is subsequently confirmed with a positive PCR test, the test centre is paid for this test; but it will not receive payment for negative tests. This incentive-compatible funding method requires no additional control measures while increasing the incentive to perform tests as diligently as possible because sloppy testing means losing money.

1 For instance, until June 2022, the German government paid testing centres a fee of €4.50 for the test material plus €8.50 for the test and for notifying the public health authority in case a test is positive; see Kassenärztliche Vereinigung Brandenburg (2022).
2 The German government, for example, spent €1 billion per month on services related to coronavirus testing (Süddeutsche Zeitung, 2022).
3 Meanwhile, the first cases of fraudulent testing in Germany have already been to court. For example, the public prosecutor's office reported that in March and April 2021 almost one million coronavirus tests were overbilled by the approximately 70 COVID-19 rapid test centres operated by the MediCan company (Burger, 2021).
4 This practice has recently been changed in Germany. With the Third Ordinance amending the Coronavirus Testing Ordinance, citizens will continue to be entitled to free testing only under certain conditions. Otherwise, they have to pay €3 for a test. We do not see how this will reduce incentives for fraudulent testing on a large scale. Moreover, with an increase in infection rates, we expect that the calls for tests free of charge will become louder.

However, the former compensation for tests, e.g. in Germany, at the rate of €11.50 would not be sufficient under this proposed scheme. Assuming that 20% of positive rapid tests prove also PCR positive and a monthly budget of, say, €460 million and 20 million tests per month, one could pay as much as €115 per confirmed positive rapid test. A further advantage of this scheme is that it can (and must) be adjusted to variations in the incidence over time and across regions. With higher (lower) incidences, the sum paid per positive test must decrease (increase) to provide proper funding of the test centres.

A variant of this financing system is to initially pay at least the material costs of €3.50 per test, in addition to the payment for confirmed positive tests. Assuming a test budget of €460 million per month, after deducting the material costs for 20 million tests per month, i.e. €70 million, €390 million would still be available to pay for rapid tests. Assuming, as above, a PCR confirmation rate of 20% per positive rapid test, €97.50 could be paid per PCR-confirmed rapid test.

A downside of this proposal might be that the number of test centres decreases and the lower number of test sites will probably decrease the number of rapid tests. With regard to the goal of detecting as many infections as possible, this would only be harmful if those who have a higher risk of infection no longer were tested. This is not expected. A high number of tests will not help fight the pandemic if they are carried out sloppily or just faked. The benefits of this proposal – a lower number of scams and sloppy testing procedures – justify the price.

Another issue might be that some of those who tested positive may approach other test centres to be tested again in order to allow them to collect another €100, in exchange for a bribe. This can be prevented by recording the payment of the fee in a central data base that is then checked for duplicate payments. Even if that is not feasible (because the government is unwilling or unable to set up such a central database), mandating a one- or two-week quarantine for those whose infection has been confirmed by a PCR test should make this kind of fraudulent behaviour too costly for most people.

### Vaccination certificates

The other issue of fraudulent behaviour concerns counterfeit vaccination records. For instance, by the end of 2021, German investigations have been ongoing against more than 11,000 people who have been accused of using counterfeit vaccination certificates (Stern, 2021). This may be just the tip of the iceberg. Since the start of the German vaccination campaign until the beginning of 2022, the number of digital vaccination certificates issued exceeds the number of vaccinations by 42.6 million (Will et al., 2022). Of course, some people were issued multiple vaccination certificates because they lost their original certificate or because they were automatically issued a certificate even though they had already obtained one. But this does not explain the enormous gap between vaccinations and issued vaccination certificates.

Indeed, counterfeit vaccination certificates are widely offered for sale on online markets. The certificates are easy to forge because they contain no security features and there is no central vaccination register. However, pharmacies are able to check via a server whether the batch number stated in the vaccination certificate really exists and has been put to use.

The economic theory of crime teaches that crime prevention requires sufficiently high penalties and a sufficiently high probability of detection. High penalties are of course cheaper; however, they are subject to limits because there is always the chance that a judicial error may occur. Therefore, one cannot rely too much on penalties and must assure a sufficiently high probability of detection.

There are not many ways to prevent the forgery of paper certificates. However, as an immediate response one could prevent the transfer of forged vaccination certificates without adequate checking of the digital vaccination pass.

A simple two-way verification procedure could work as follows:

- The person to be vaccinated has to prepare two pre-stamped envelopes. One envelope is addressed to the doctor (or vaccination centre), the other to the vaccinated person.
- The doctor vaccinates and records the kind and date of the vaccination in the vaccination certificate, hands it over to the vaccinated person and keeps the letter to the vaccinated person on file.
- The vaccinated person presents the vaccination certificate and his or her identification to the pharmacist and hands over the pre-stamped letter addressed to the doctor. The pharmacist generates the barcode and mails it in the pre-stamped letter to the doctor.
- The doctor forwards the barcode in the pre-stamped letter to the vaccinated person.

This procedure does not require significant effort. However, the pharmacy has to verify the doctor's name and address. Statutory health insurance physicians are registered with the health insurance organisation. However, private doctors are not, which may be a problem. But generally, even a simple Google search may be sufficient.

In a completely digitised society, another method to fight fraud is feasible, but more complex as it relies on some asymmetric cryptographic techniques. The main feature of those techniques is that there is a public and a private key to encrypt or decrypt data. The private key is in the possession of the owner and not shared with the public. The public key is shared with the public, i.e. everyone can use it to code or decode messages to and from the owner of the private key, that person being the only one able to decrypt data encrypted by means of the public key. This principle also works the other way around: The owner of the private key encrypts a message or data by means of the private key, which also creates a corresponding pubic key. This message can only be decrypted by using the corresponding public key; any message that cannot be decrypted by the public key is not from the owner of the corresponding private key.

By using this technology, a procedure to reduce the number of forged vaccination certificates reads as follows:

- The doctor generates a private and a public key; the public key is shared with the public (e.g. stored on a public server), the private key is only known to the doctor.
- After vaccinating a patient, the doctor generates a barcode that contains personal data and vaccination data of the patient, using the private key.
- To control the vaccination status of a person, everybody can decipher the barcode by using the public key. If the barcode cannot be decrypted by means of the public key, the code is forged.

The advantage of this procedure is that no central vaccination register is required, and there is no data protection problem. However, vaccination certificates can still be forged by dishonest doctors.

## Inefficient testing

Another failure of the containment policy concerns the lack of sufficient testing capacity, aggravated by inefficient testing methods. This problem was particularly severe in Germany and England (BBC, 2021). Of course, widespread testing combined with following up the chains of infections is essential for the containment of a pandemic. Some other countries seem to have sufficient testing capacities. Austria is a European country that did a better job in testing. For example, in Vienna PCR mouth rinse/gargle tests are available free of charge for anyone who lives, works or attends school in Vienna. People register online, receive a barcode and pick up a test kit at a drugstore or pharmacy. After performing the test at home, people deliver the test to a supermarket, gas station or drugstore that hands in the sample to the laboratory, which uses pooling methods. This makes those tests fairly cheap. In fact, the Viennese laboratory operator Lifebrain charges only €6 per test, in sharp contrast to Germany, where prices in the range of €70 and higher are common (Macho and Salz, 2022). According to the co-manager of Lifebrain, it is the pooling method that saves resources and makes the test cheap. Other contributing factors are economies of scale, centralisation and automation.

Figure 2

**New coronavirus tests per 1,000 inhabitants in selected countries (smoothed), December 2021-January 2022**

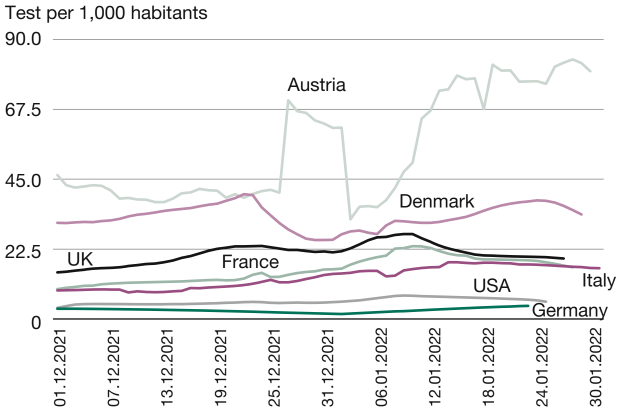

Source: Our World in Data (2022), own depiction.

Figure 2 shows how the number of new tests per 1,000 inhabitants varied across selected countries (Austria, Denmark, France, Italy, Germany, the UK and the USA). Among these countries, Germany carried out the lowest number of tests per 1,000 inhabitants. Germany also failed to install sufficient testing capacity and failed to follow-up the chains of infections. Figure 3 displays the testing capacities per day in Germany.

Instead of testing each individual sample, one can instantly increase the capacity for testing by testing pools of samples. A simple pooling procedure involves taking two samples per person and combining one of the two samples in a pool and testing the pool. If the pool tests negative, all samples within that pool must be negative as well. If the pool tests positive, all members of that pool are tested individually using the second sample. Pooled testing works because the pathogen shows up equally in individual and in pooled samples. However, the pool should not be too large, because otherwise a loss of sensitivity associated with sample dilution may become critical.

Figure 3
**Test capacity in Germany**

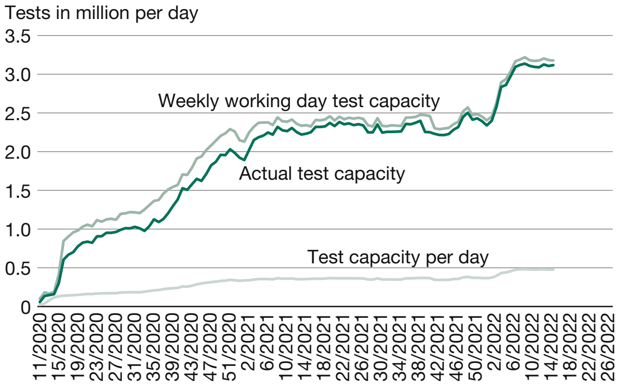

Note: By calendar week and year.

Source: RKI (2022).

In Germany, the testing of pooled samples has been used since the 1990s to test donated blood for HIV and hepatitis viruses. Currently, it is also applied to test for infections with the coronavirus in schools and childcare centres (Schlenger, 2020; Koestler, 2021). The pooling method is also suitable for testing employees in a company, students, and staff in schools, hospitals, as well as nursing home staff and inhabitants.

Pooling is far more efficient than the exclusive testing of individual samples (Dorfman, 1943; Gollier and Gossner, 2020). However, one can considerably improve the testing of pooled samples with the following method that makes use of binary codes (Wolfstetter, 2022).

We explain the proposed pooling method with a simple example. Suppose a group of seven people {1,2,3,4,5,6,7} shall be tested. In the first step, one represents each person's number by its binary code, and then proceeds to test an intelligently designed collection of pooled samples. Binary codes represent data using a sequence of binary symbols, typically 0 and 1. Binary codes are concise and efficient. For example, a binary code of length 3 allows the unique representations of the integers 1 to 7, length 5 to 31, length 10 to 1,023, and length 15 to 32,767. The binary codes that represent the seven people to be tested are:

$$1 \rightarrow (001),\ 2 \rightarrow (010),\ 3 \rightarrow (011),\ 4 \rightarrow (100),$$
$$5 \rightarrow (101),\ 6 \rightarrow (110),\ 7 \rightarrow (111).$$

There, the last 1 represents $2^0 = 1$, the second last $2^1 = 2$ and the first $2^2 = 4$, and the reverse mapping from binary code to person is defined as:

$$(001) \rightarrow 1\times2^0 + 0\times2^1 + 0\times2^2 = 1$$

$$(010) \rightarrow 0\times2^0 + 1\times2^1 + 0\times2^1 = 2$$

$$(110) \rightarrow 0\times2^0 + 1\times2^1 + 1\times2^2 = 6$$

$$(111) \rightarrow 1\times2^0 + 1\times2^1 + 1\times2^2 = 7.$$

In general, the binary code of length L: $(v_1, v_2 \ldots v_{(L-1)}, v_L)$ represents $\sum_{i=0}^{L-1} v_{L-1} 2^i$ persons, where each $v_i$ is either zero or one, and at least one $v_i$ is equal to one. Having represented persons by the binary codes, one applies the following testing procedure:

- Take samples from each of the seven people and split each into four copies. (Keep one additional copy on reserve in case one also considers sub-pools.)
- Deposit a copy of each person's sample into that person's test tube and copies into pooled test tubes (called pools).
- Specifically, put a copy from the sample of each person with a 1 at the last digit of their binary code into pool 1, a copy from the sample of each person whose binary code has a 1 at the second last place into pool 2, and a copy from the sample of each person who has a 1 at the first place into pool 3.
- Test each of the three pools and record which pool(s) tested positive.

Note that copies of a person's sample may be included in several pools. For example, copies of the sample of person 3, with binary code (011), are included in pools 1 and 2, and those of person 7 are included in all three pools, whereas those of persons 1, 2 and 4 are included in only one pool each.

Having tested the three pools, the infected persons are identified as follows: If only one pool tested positive, say pool 1, then the one person with binary code (001), and no one else, is infected. In that case, one has uniquely detected who is infected. The important observation is that no person with a 1 in positions other than the last place of its binary code is infected. This drives the conclusion that person 1 with binary code (001) must be the one and only one who is infected.

In this case, only three tests (the tests of three pools) are needed to detect who is infected, whereas the standard method of testing all persons would include performing seven tests, and the standard method of testing one pool would include performing eight tests (one test of the pool, followed by seven individual tests, after having observed that the pool tested positive).

If two pools tested positive, say pool 1 and 2, one knows that all persons with a 1 in the first place of their binary code cannot be infected. Therefore, the infected person(s) must be among those with binary codes {(001), (010), (011)}. In other words, the infected person(s) must be among persons {1,2,3}. In that case, one could continue to test these three persons. However, one can do better, for example, by testing the sub-pool of persons with binary codes {(010), (011)}[5]. If this sub-pool tests negative, one can infer that person 1 and no one else is infected; only if this sub-pool tests positive would one have to test all three persons. In that case, the infected person(s) are identified in at least four and at most six tests, whereas the standard method of testing all persons' samples would have performed seven tests and the standard pooling method eight tests.

If all three pools tested positive, one could continue and test all seven persons. However, one can do better, for example by testing the sub-pool of persons {3, 5, 6, 7}, with binary codes {(011), (101), (110), (111)}. If this sub-pool tests negative, one can infer that persons {1, 2, 4}, with binary codes {(001), (010), (100)} must be infected, and no one else; only if this sub-pool tests positive, would one have to test more persons.

Finally, if all pools tested negative, one concludes that no person is infected. In that case, the standard method of testing all individual samples would have performed seven tests and the standard pooling method would have performed one test, whereas the proposed pooling method performed three tests. Therefore, if it is highly likely that no person is infected, the standard pooling method performs best. We conclude that the proposed pooling method is more efficient than the standard methods, except if it is most likely that either all persons are infected, in which case it is best to simply test all persons' samples, or no person is infected, in which case it is best to employ the standard pooling method.

We mention that the standard pooling method is not effective if one tests in hot spots with high infection rates. For example, if infection rates are around 30% or higher, the probability that the pool tests positive, and subsequently all individuals have to be tested, is close to one. This can be seen in Table 1, which displays the probability that the pool tests positive, depending on the size of the pool n, and the infection rate x. The standard pooling method has been widely used in Austria with a pool size of n = 10.[6]

5 However, this requires that one splits persons' individual samples into more than four copies.

6 The probability that the pool tests positive is equal to $1 - (1 - x)^n$, assuming infections are independent events. This assumption is, however, too restrictive if the members of the tested group have been in close contact with each other.

Table 1
**Probability that a pool tests positive depending on the infection rate**

| | Infection rate (x) | | |
|---|---|---|---|
| Pool size (n) | x = 0.1 | x = 0.3 | x = 0.5 |
| 10 | 0.651322 | 0.971752 | 0.999023 |
| 11 | 0.686189 | 0.980227 | 0.999512 |
| 12 | 0.71757 | 0.986159 | 0.999756 |
| 13 | 0.745813 | 0.990311 | 0.999878 |
| 14 | 0.771232 | 0.993218 | 0.999939 |
| 15 | 0.794109 | 0.995252 | 0.999969 |

Source: Own calculations.

If one tests larger groups of people, the expected saving from using the proposed pooling method is increasing, although designing an efficient sequential procedure to detect who is infected becomes more complex. However, one could write a computer programme that provides complete instructions to those who run the tests. Equipped with such a programme, running the tests requires no understanding of the complexity of the procedure. Finally, we mention that the proposed pooling procedure is particularly efficient if one happens to know how many people are infected. In particular, if one knows that only one person is infected, one knows immediately who is infected after testing all three pools. For example, if only pools 2 and 3 test positive, one can infer immediately that person 3, with binary code (011), is infected.

## Conclusion

In this short article, four pressing issues of the fight against the coronavirus pandemic are discussed from an economic perspective: the vaccination gap, test-billing and vaccination fraud, as well as the shortage of testing capacity, aggravated by inefficient testing methods.

If one wants to close the vaccination gap, a vaccination register is required. To make such a register safe for falsifications, a two-sided mechanism is proposed that makes sure that only real vaccinations are documented. As this mechanism is not completely foolproof, it cannot be completely guaranteed against criminal infringements.

The billing of rapid antigen coronavirus tests suffers from its ineffectiveness, as well as from its economic inefficiency. The reason for these tests is to discover coronavirus infected persons as quickly as possible and to check positive tests with a more accurate PCR test. Economically, the detection of infected persons should be incentivised and documented by a PCR test. Therefore, it is proposed to pay rapid antigen corona tests (beside the payment for test material) only if a positive

PCR test is documented in the respective cases. However, the payment per positive PCR test must be set sufficiently high.

With restricted capacities for PCR tests, more sophisticated test strategies are required. Pool tests are already common in Germany. In this paper, a refinement of pool testing with binary codes is proposed that increases the PCR testing capacity considerably.

Even if the new mechanisms proposed in this paper may not be applied in the coronavirus pandemic, they could be implemented in advance for similar future crises. As the expression goes, prevention is the best medicine.

## References

[CR1] BBC (2021, 14 December), Covid-19: High demand blamed for shortage of PCR appointments in England, https://www.bbc.com/news/uk-59651166 (25 August 2022).

[CR2] Beck, H., A. Prinz and E. Wolfstetter (2021, 13 December), 115 Euro pro Positiv-Test statt 11,50 Euro für jeden — so lassen sich Betrüger stoppen, *Welt*, https://www.welt.de/wirtschaft/article235628926/Betrugmit-Corona-Tests-Man-sollte-nur-positive-Ergebnisse-vergueten.html (25 August 2022).

[CR3] Burger, R. (2021, 2 December), MediCan: Prozess um mutmaßlich massenhaften Betrug bei Corona-Test. *Frankfurter Allgemeine*, https://www.faz.net/aktuell/gesellschaft/kriminalitaet/medican-prozess-bei-980-000-corona-tests-zu-viel-abgerechnet-17663478.html (3 December 2021).

[CR4] Dorfman R (1943). The Detection of Defective Members of Large Populations. Annals of Mathematical Statistics.

[CR5] Gollier C, Gossner O (2020). Group Testing against Covid-19. Econ-Pol Policy Briefs.

[CR6] Kassenärztliche Vereinigung Brandenburg (2022), Testungen auf SARS-CoV-2 im Überblick (Update), https://www.kvbb.de/praxis/service/ansicht-news/article/testungen-auf-sars-cov-2-im-ueberblick-update/1/ (25 August 2022).

[CR7] Koestler, C. (2021, 20 December), Tücken bei den Pool-Tests, *Süddeutsche Zeitung*, https://www.sueddeutsche.de/muenchen/wolfrat-shausen/pool-test-coronavirus-schule-bad-toelz-wolfratshausen-pandemie-1.5491922 (25 August 2022).

[CR8] Macho, A. and J. Salz (2022, 25 January), Warum hat Österreich reichlich günstige PCR-Tests, Deutschland aber nicht?, *WirtschaftsWoche*, https://www.wiwo.de/unternehmen/dienstleister/coronatests-warumhat-oesterreich-reichlich-guenstige-pcr-tests-deutschland-abernicht/28004238.html (25 August 2022).

[CR9] Our World in Data (2022), Covid-19 Data Explorer, https://ourworldindata.org/explorers/coronavirus-data-explorer (19 September 2022).

[CR10] RKI (2022), Digitales Impfquotenmonitoring zur COVID-19-Impfung, https://www.rki.de/DE/Content/InfAZ/N/Neuartiges_Coronavirus/Daten/Impfquoten-Tab.html (19 September 2022).

[CR11] Schlenger R (2020). Pooling bei Coronatests: Eine Lösung mit Tücken. Deutsches Ärzteblatt.

[CR12] Stern (2021, 26 December), Massenhaft gefälschte Impfpässe: Mehr als 11.000 Ermittlungen laufen, *Stern*, https://www.stern.de/politik/massenhaft-gefaelschte-impfpaesse—-mehr-als-11-000-ermittlungenlaufen-31454432.html (25 August 2022).

[CR13] Süddeutsche Zeitung (2022, 24 June), Bürgertests sollen künftig drei Euro kosten, *Süddeutsche Zeitung*, https://www.sueddeutsche.de/politik/corona-lauterbach-buergertests-1.5608623 (25 August 2022).

[CR14] Will, S., L. Landes, S. Gubernator and L. Pfahler (2022), RKI meldet 203.136 Neuinfektionen — Inzidenz steigt auf 1017, 4, *Welt*, 27.01.2022, https://www.welt.de/vermischtes/article236506265/Corona-RKI-meldet-203-136-Neuinfektionen-Inzidenz-steigt-auf-1017-4.html.

[CR15] Wolfstetter, E. (2022), Efficient Pooling in Covid-19 Testing, Working Paper.

